# The Roles of Neutrophils Linking Periodontitis and Atherosclerotic Cardiovascular Diseases

**DOI:** 10.3389/fimmu.2022.915081

**Published:** 2022-07-07

**Authors:** Rizky A. Irwandi, Scott T. Chiesa, George Hajishengallis, Venizelos Papayannopoulos, John E. Deanfield, Francesco D’Aiuto

**Affiliations:** ^1^ Periodontology Unit, UCL Eastman Dental Institute, University College London, London, United Kingdom; ^2^ UCL Institute of Cardiovascular Science, University College London, London, United Kingdom; ^3^ Department of Basic & Translational Sciences, Laboratory of Innate Immunity & Inflammation, Penn Dental Medicine, University of Pennsylvania, Philadelphia, PA, United States; ^4^ Antimicrobial Defense Laboratory, The Francis Crick Institute, London, United Kingdom

**Keywords:** neutrophils, systemic inflammation, trained immunity, innate immune memory, periodontitis, periodontal disease, atherosclerosis, atherosclerotic cardiovascular disease

## Abstract

Inflammation plays a crucial role in the onset and development of atherosclerosis. Periodontitis is a common chronic disease linked to other chronic inflammatory diseases such as atherosclerotic cardiovascular disease (ASCVD). The mechanistic pathways underlying this association are yet to be fully understood. This critical review aims at discuss the role of neutrophils in mediating the relationship between periodontitis and ASCVD. Systemic inflammation triggered by periodontitis could lead to adaptations in hematopoietic stem and progenitor cells (HSPCs) resulting in trained granulopoiesis in the bone marrow, thereby increasing the production of neutrophils and driving the hyper-responsiveness of these abundant innate-immune cells. These alterations may contribute to the onset, progression, and complications of atherosclerosis. Despite the emerging evidence suggesting that the treatment of periodontitis improves surrogate markers of cardiovascular disease, the resolution of periodontitis may not necessarily reverse neutrophil hyper-responsiveness since the hyper-inflammatory re-programming of granulopoiesis can persist long after the inflammatory inducers are removed. Novel and targeted approaches to manipulate neutrophil numbers and functions are warranted within the context of the treatment of periodontitis and also to mitigate its potential impact on ASCVD.

## Introduction

Atherosclerotic cardiovascular disease (ASCVD) consists of a group of disorders that affect the heart and blood vessels (1) and include coronary heart disease, cerebrovascular disease, and peripheral vascular disease (2). ASCVD is a major cause of global mortality and a leading contributor to disability as it causes 18.6 million deaths and contributes to 34.4 million people living with disability in 2019 (3). Although its pathogenesis, progression, and complications comprise multiple complex processes, inflammation plays a key role in each stage of the disease (4).

Periodontitis is a common chronic inflammatory disease caused by oral microbial dysbiosis. The onset and progression of the disease could span over decades and is influenced by genetic and environmental factors. This prevalent oral disease is characterized by progressive destruction of hard and soft tissues supporting the tooth, including the periodontal ligament and alveolar bone (5). Untreated periodontitis leads inevitably not only to tooth loss but also to masticatory impairment and negative influences on the quality of life of a patient (6). Like ASCVD, periodontitis is a major public health concern as it affects over half of the population of the world (7) and 5–15% of the global population presents a severe form of the disease (8), causing increased costs of oral healthcare (9).

The evidence linking periodontitis to systemic diseases has previously focused on the findings that periodontal bacteria and their endotoxins disseminate physically through the blood circulation (10, 11). However, periodontitis also triggers systemic inflammation, indicated by an increased level of C-reactive protein (CRP), TNFα, IL-1β, and IL-6 in the serum of patients (2). Because of chronic inflammation occurring at the periodontium, endotoxemia, bacteremia, and systemic inflammation are collectively implicated in numerous systemic diseases, including atherosclerotic cardiovascular disease (ASCVD) (12–14).

Neutrophils are the most abundant inflammatory cells in humans and the first-line defense against infection in the innate-arm of the immune system. They are derived from the myeloid differentiation lineage of hematopoietic stem cells (HSCs) in the bone marrow. Upon detection of pathogens, neutrophils capture and destroy invading pathogens *via* phagocytosis and intracellular degradation, degranulation, and the formation of neutrophil extracellular traps (NETs) (15). Moreover, the emerging evidence over the past decade reveals that neutrophils are involved in chronic inflammation and are implicated in chronic inflammatory disorders, including periodontitis and ASCVD (16–20). Periodontitis appears to be associated with hyper-responsive neutrophils (21–23), which might, at least in part, be attributed to the notion that oral disease could influence hematopoietic tissue activity and trained immunity (12). Trained immunity represents a non-specific memory in innate immune cells that is induced by earlier encounters with infectious or inflammatory stimuli and which promotes increased immune responses to future challenges with the same or different stimuli (24, 25). Meanwhile, in ASCVD, neutrophils contribute to different stages and clinical manifestations of atherosclerosis (26) and literature also suggests that inflammation-adapted hematopoietic stem and progenitor cells (HSPCs) may contribute to the disease pathogenesis (27–29). As such, recent consensus between the European Federation of Periodontology and the World Heart Federation includes neutrophil hyper-responsiveness as one of the mechanisms to explain the epidemiological association between periodontitis and ASCVD (2).

Mechanisms linking periodontitis to ASCVD and the effect of periodontitis treatment in improving the surrogate markers of ASCVD in an attempt to show causal interactions between the two diseases have been extensively explored (2, 14). However, the causal mechanistic pathways between these two common non-communicable diseases are yet to be fully understood. In this review, we aim to critically address the role of neutrophils in linking periodontitis to ASCVD. Systemic inflammation triggered by different causes, including periodontitis, may drive inflammatory adaptation of HSPCs and trained granulopoiesis in the bone marrow, resulting in increased production of neutrophils with a hyper-responsive phenotype (12, 30). This systemic inflammation-driven modification of granulopoiesis can contribute to atherosclerosis in a stage-dependent manner. However, although the periodontitis treatment successfully achieves the resolution of the periodontal tissue site and improves surrogate markers of ASCVD, studies reveal that the hyper-responsive function in neutrophils may persist (23, 31). Therefore, novel approaches to target neutrophils by manipulating their numbers and functions are warranted in periodontitis treatment and to mitigate its impact on ASCVD.

## The Link Between Periodontitis and ASCVD

The impact of the treatment of periodontitis on cardiovascular outcomes and surrogate markers of ASCVD has been extensively explored. Patients with periodontitis exhibit an increased risk of coronary and cerebrovascular events compared with periodontally healthy individuals. These findings may not apply to the whole population as influenced by the demographic characteristics, individuals, studies, and case definition of periodontitis (2, 32). In the ARIC study on 6736 dentate participants with 299 incidents of ischemic stroke, it was revealed that seven periodontal profile classes, that were used to assess the participants were associated with an increased risk of cardioembolic and thrombotic stroke subtypes compared with periodontally healthy participants. The assessment in this study was based on seven tooth-clinical parameters, resulting in seven different periodontal profile classes, and the greater class indicates a more severe form of periodontitis (33). Lastly, based on the 1999–2010 Taiwanese National Health Insurance Research Database involving 393,745 patients with periodontitis and 393,745 non-periodontitis individuals, there was a significantly increased incidence of arterial fibrillation in patients with periodontitis compared with controls (34). The findings from all studies had been adjusted for a wide range of potential confounders, indicating that periodontitis is an independent risk factor for an increased risk of ASCVD events.

Treatment of periodontitis may influence the progression of ASCVD. The study involving 511,630 periodontitis patients and 208,713 individuals without periodontitis from The Longitudinal Database of Taiwan’s National Health Insurance demonstrated that patients receiving dental prophylaxis had a lower hazard ratio of acute myocardial infarction compared to periodontally healthy controls, suggesting an almost 10% reduction in the risk of a new ASCVD event (35). These improvements were not observed across all types of periodontitis and controls, suggesting a difference in host susceptibility and response even after the treatment of peridontitis when looking at future incidence of ASCVD events (36).

Consistent evidence suggests that periodontitis is associated with higher blood pressure and endothelial dysfunction (2, 37–39). A recent review and subsequent meta-analysis of intervention studies confirmed that the management of periodontitis can be a novel non-drug approach for the treatment of hypertension. The evidence is still inconclusive as to whether the treatment of periodontitis influences blood pressure even in the absence of hypertension (40). The basis of this improvement in vascular function could be linked to the effect of the treatment of periodontitis in improving endothelial function as assessed by flow-mediated dilation (FMD) (41–44). A possible mechanism by which local periodontal treatment improves endothelial function in periodontitis patients is endothelial nitric oxide synthase/nitric oxide (eNOS/NO) activation. NO is mainly produced by eNOS in endothelial cells and induces the relaxation of smooth muscle cells (45, 46). Meanwhile, IL-6, TNFα, IL-1β, and CRP directly reduced eNOS at both mRNA and protein levels in human endothelial cells (47–49). As a result, NO bioavailability in the serum is reduced, leading to endothelial dysfunction and periodontitis is associated with this surrogate marker of ASCVD (50, 51). Hepatocytes are the major producers of CRP triggered by IL-6 and IL-1β stimulation, while TNFα also upregulates CRP production in human coronary artery smooth muscle cells (52–55). A recent meta-analysis reveals a progressive CRP level reduction up to 6 months in patients with periodontitis following effective treatment (37). This was also associated with a reduction of reduced serum IL-6, TNFα, and IL-1β in patients with periodontitis compared to baseline (56–59). Collectively, these findings suggest that reduced levels of CRP, IL-6, TNFα, and IL-1β after treatment of periodontitis could restore eNOS activity and NO bioavailability, resulting in improved endothelial dysfunction.

## Periodontitis as a Trigger of Systemic Inflammation

Periodontitis and ASCVD share similar hallmarks of inflammatory mechanisms (60), genetic (2, 61), and common risk factors (62). However, a significant body of evidence supports an independent association between periodontitis and ASCVD following adjustment for confounders and shared risk factors (63, 64). This independent association can be explained by the capability of periodontitis to trigger a low-grade but consistent systemic inflammation, which may contribute to the development of ASCVD (65). Patients with periodontitis exhibit an elevated level of systemic pro-inflammatory mediators, which include CRP, TNFα, IL-1β, IL-6, as well as increased neutrophil numbers in the blood (2, 14, 66–69). A retrospective study involving 60,174 participants revealed that even after the adjustment of confounders, participants with periodontitis were 1.59 times more likely to have ASCVD (63). Previously, an 8-year prospective cohort study involving 11,869 participants also showed that those reporting poor oral hygiene had an enhanced risk of CVD events as well as an elevated level of CRP and fibrinogen in the serum (70).

Systemic inflammation triggered by periodontitis potentially occurs because of bacterial dissemination or periodontal tissue-derived inflammatory mediator leakage into the blood circulation. The ulceration of the epithelium owing to local periodontal inflammation along with the support of its rich vascularization may provide greater access for bacteria and their endotoxins such as lipopolysaccharides (LPS) to the circulation, leading to bacteremia (13). This event has been reported in patients with periodontitis during mastication, toothbrushing, and dental scaling (10, 11). Bacteremia would induce inflammatory alterations in the endothelium, which include enhanced expression of adhesion molecules and the production of pro-inflammatory cytokines. With regard to the spillover of inflammatory mediators into the bloodstream, this can affect vascular tissues as well as other distant organs, including the liver, which then may initiate an acute-phase response (14).

## Inflammation and Neutrophils: Implications for Atherosclerosis

**Figure 1:**
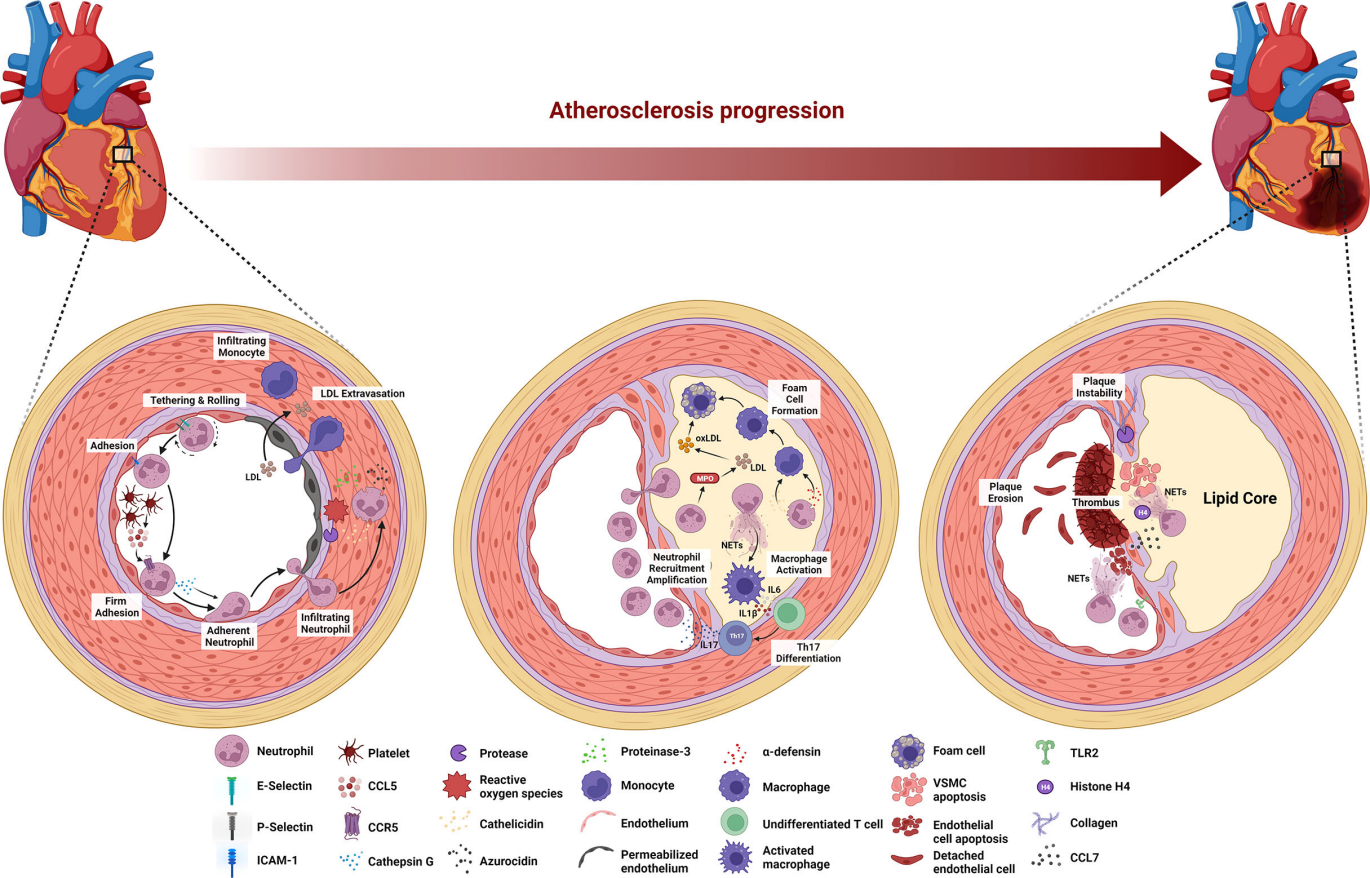
Stage-dependent role of neutrophils in atherosclerosis. During the early stage of atherosclerosis, upregulation of E-selectin, P-selectin, and ICAM-1 induces neutrophil recruitment. Platelet-derived CCL5 activates neutrophils to release cathepsin G, leading to the firm adhesion to and accumulation of neutrophils in the endothelium. ROS and proteases secreted by neutrophils activate and dysregulate the endothelial cell layer and degrade the underlying extracellular matrix, resulting in monocyte infiltration and LDL extravasation. Neutrophils secrete MPO that mediates LDL oxidation and promotes foam cell formation. Cathelicidin- and α-defensin-derived neutrophils activate macrophages towards pro-inflammatory state, while NETs stimulate macrophage to release IL-6 and IL-1β that promote Th17 differentiation followed by the amplification of neutrophil recruitment. At the late stage of atherosclerosis, activated VSMCs induce neutrophil chemotaxis and release CCL7 to stimulate NETs. Histone H4-derived from NETs induces VSMC lysis and the secretion of proteases by neutrophils degrades collagen and lyses VSMCs, leading to the plaque instability. Neutrophils contribute to plaque erosion through NET release as well as colocalization with TLR2 of endothelial cells to induce endothelial cell stress and apoptosis.

Atherosclerosis is a cause of myocardial infarction, ischemic cardiomyopathy, and ischemic stroke that contribute to most deaths in the population worldwide (4). Arterial wall damage due to blood lipid profile imbalance, oscillating shear stress, and pro-inflammatory mediators initiates this underlying ASCVD pathology. The subsequent processes are endothelial cell activation in arterial tissue, myeloid cell adhesion to the endothelium, and infiltration into the arterial intima (26). At the late stage of atherosclerosis, inflammatory cell accumulation, lipoprotein deposition, and cellular debris buildup are responsible for the arterial plaque formation followed by the plaque instability leading to atherosclerotic plaque rupture. Atherosclerosis is a decades-long process with many stages of evolution, and evidence suggests that neutrophils are involved in different stages of atherosclerosis (**Figure 1**) (26).

The onset of atherosclerosis is characterized by endothelial dysfunction, which induces neutrophil recruitment to the endothelium. Specifically, the dysfunction up-regulates the expression of various endothelial cell adhesion molecules, including E-selectin, P-selectin, and intracellular adhesion molecule-1 (ICAM-1) (71). Platelets then deliver CCL5, the primary ligand of CCR5 on the endothelium and promote cathepsin G secretion by neutrophils, resulting in the firm adhesion to and accumulation of the cells in the endothelium (72, 73). Furthermore, neutrophils aggravate endothelial dysfunction by secreting reactive oxygen species (ROS), azurocidin, proteinase 3, cathelicidin, and cathepsin G in the arterial lumen (26, 74). ROS and proteases activate and dysregulate the endothelial cell layer and degrade the underlying extracellular matrix, enabling leukocyte infiltration and low-density lipoprotein (LDL) extravasation (26). Azurocidin also contributes to increasing endothelial permeability (26, 74–76), whereas azurocidin, proteinase 3, cathelicidin, α-defensin, and cathepsin G all promote myeloid cell recruitment and facilitate monocyte entry into the atherosclerotic lesions (74, 77–82).

The progression of the lesion continues following the aggravation of endothelial dysfunction by neutrophils, where macrophage activation and foam cell formation occur in the arterial intima. Neutrophil-derived granule proteins, cathelicidin, and α-defensin activate macrophages toward a pro-inflammatory state (M1 macrophage phenotype). Neutrophils also secrete myeloperoxidase (MPO) to generate oxygen radicals that oxidize apolipoprotein B, a protein structure in LDL (82). Subsequently, macrophages take up this oxidized LDL (oxLDL), resulting in foam cell formation (83). Moreover, NETs stimulate macrophages by turning on transcription factors encoding IL-6 and IL-1β. These cytokines promote the differentiation of Th17 cells, which in turn amplify neutrophil recruitment in the lesion (84). At this stage, because of sustained myeloid cell recruitment and foam cell generation, the atheroma becomes pronounced.

In the late stage of atherosclerosis, neutrophils destabilize the atherosclerotic plaque. Mechanistically, activated vascular smooth muscle cells (VSMCs) in advanced atherosclerotic lesions induce neutrophil chemotaxis and secrete CCL7, which stimulates NET release. One of the NET cytotoxic components, histone H4, disrupts the integrity of the VSMC plasma membrane, leading to cell lysis (85). Moreover, endotoxemia in a mouse model of atherosclerosis revealed that leukotriene B4-induced neutrophil recruitment to atherosclerotic plaques induces collagen degradation and VSMC lysis, leading to the feature of plaque instability (86). In eroded human plaques, neutrophils colocalized with toll-like receptor 2 of endothelial cells, and an *in vitro* experiments showed that co-culture of neutrophils with endothelial cells potentiates endothelial stress and apoptosis, resulting in endothelial cell detachment followed by luminal endothelial cell desquamation (plaque erosion) (19). Lastly, NETs are also involve during endothelial erosion as the disruption of NETs by either peptidyl arginine deiminase 4 (PAD4) gene knockout or DNase I treatment in atherosclerotic mice attenuates endothelial disintegration and endothelial cell apoptosis (87).

**Figure 2:**
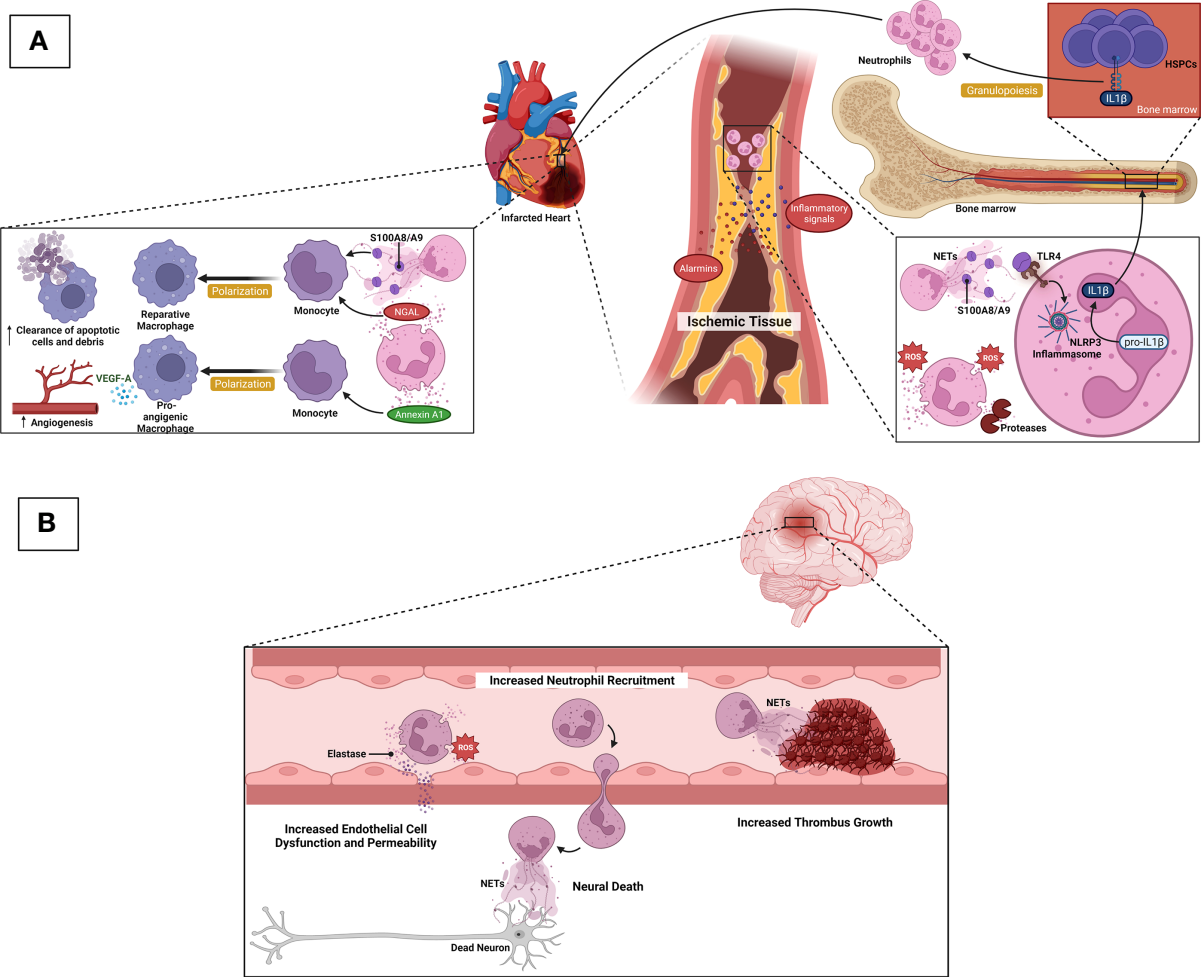
Neurophils in ASCVD complications. **(A)** After acute myocardial infarction, ischemic/necrotic tissues release alarmins and inflammatory signals to induce neutrophil recruitment. Activated neutrophils secrete ROS, proteases, and NETs. S100A8/A9-derived from NETs induces IL-1β release following NLRP3 inflammasome priming in naïve neutrophils. IL-1β reaches the bone marrow to stimulate granulopoiesis leading to the amplification of neutrophil production and accumulation, which in turn, detriments the ischemic heart and eventual heart failure. During cardiac tissue repair, NGAL and S100A8/A9 stimulate macrophages to shift towards reparative phenotypes, resulting in increased clearance of apoptotic cells/debris. Neutrophils also secrete Annexin A1 to favor the shift of macrophages towards pro-angiogenic phenotypes that release VEGFA to promote angiogenesis in the ischemic site of cardiac tissue. **(B)** Following ischemic stroke, dying neurons attract neutrophils to the ischemic area, and neutrophil ROS and elastase promote endothelial cell dysfunction and vascular permeability. NETs contribute to thrombus growth, thereby increasing stroke volume. Neutrophils also promote neuronal cell death that is likely mediated by NETs.

Neutrophils play dual roles, which are both adverse and favorable for cardiac tissue repair following myocardial infarction (**Figure 2A**). Tissue necrosis/ischemia post-acute myocardial infarction releases alarmins and inflammatory signals that attract neutrophils to the site of infarction (88). Activated neutrophils secrete ROS, proteases, NETs, and IL-1β. Granulopoiesis stimulated by IL-1β leads to neutrophil accumulation in the injured site, which is harmful to the remodeling of the ischemic area, resulting in eventual heart failure (89, 90). Following neutrophil accumulation, monocytes and monocyte-derived macrophages infiltrate the infarcted site to phagocytose cell debris and apoptotic neutrophils, activating cardiac repair (88). Intriguingly, neutrophils also contribute to this cardiac healing as their damage-associated molecular patterns (DAMPs), such as neutrophil gelatinase-associated lipocalin (NGAL) and S100A8/A9, stimulate macrophages to shift toward reparative phenotypes (91, 92). These anti-inflammatory macrophages aid in the resolution of inflammation (88). Neutrophils also secrete annexin A1, which stimulates pro-angiogenic macrophage polarization. These macrophage phenotypes release vascular endothelial growth factor A (VEGFA) to support angiogenesis in the ischemic site of the myocardium (93). Besides cardiac tissue repair, neutrophils aggravate other atherosclerosis complications, namely, ischemic stroke (**Figure 2B**). Following an episode of ischemic stroke, dying neurons attract neutrophils to the area to release ROS and elastase to enhance endothelial cell dysfunction and permeability. NETs promote thrombus growth, thereby increasing stroke volume. Finally, neutrophils increase neuronal cell death in a process that is likely to involve NETs (26, 94).

## Impact of Periodontitis-Induced Systemic Inflammation on Bone Marrow Activity and Subsequent Circulating Neutrophil Alterations: Plausible Mechanisms

### Modulation of HSPCs in the Bone Marrow: A Key Role for Periodontitis-Induced Systemic Inflammation

Trained immunity can be initiated in the bone marrow *via* sustained epigenetic, metabolic, and transcriptional adaptations in HSPCs, leading to enhanced myeloid-biased differentiation and production of increased numbers of trained myeloid cells, including neutrophils (trained myelopoiesis/granulopoiesis) (95, 96). This training is based on the ability of HSPCs to sense numerous inflammatory cues in response to hematopoietic stress such as systemic inflammation (12, 96). HSPCs can directly sense pathogen-associated molecular patterns (PAMPs), such as LPS, *via* their pattern recognition receptors (PRRs), such as toll-like receptors (for example, TLR-4 for sensing LPS). Regarding the direct mechanism, in the context of periodontitis-induced bacteremia, it could be envisioned that systemically disseminated periodontal pathogens or their products (for example, LPS or lipopeptides) may also reach the bone marrow, resulting in innate immune training of HSPCs. However, indirect activation relies on specialized cells residing in either the peripheral tissue or bone marrow to affect the hematopoietic system through the release of cytokines (97, 98).

In addition to direct sensing of pathogens, cytokines and growth factors derived from both the bone marrow niche and peripheral tissue can mediate indirect adaptation of HSPCs (97, 98). IL-1β promotes myeloid differentiation and self-renewal of HSPCs as chronic administration of this cytokine elevates the number of myeloid-bias HSPCs. This is also consistent with the recent finding that enhanced myelopoiesis of HSPCs in a mouse model of β-glucan or experimental periodontitis-induced trained immunity is mediated by IL-1β (95, 99). TNFα exhibits different actions in HSPCs as it promotes both the survival and myeloid differentiation of HSCs while inducing the apoptosis of myeloid progenitors (100). The increased level of IL-6 in the bone marrow niche promotes myelopoiesis, indicated by an elevated number of multipotent progenitors (MPPs) and common myeloid progenitors (CMPs) (101). Type 1 IFN mediates trained granulopoiesis in mice following β-glucan treatment resulting in the production of neutrophils with an enhanced ROS-dependent anti-tumor phenotype (102). IFNγ promotes HSC self-renewal and myeloid differentiation in a mouse model of repeated *Mycobacterium avium* infection (103). Granulocyte colony-stimulating factor (G-CSF) is a key growth factor that drives granulopoiesis. Specifically, G-CSF produced by monocytes in the bone marrow niche is responsible for HSPC mobilization from the bone marrow to the circulation (104). Moreover, in emergency granulopoiesis, G-CSF promotes the expansion and granulocyte lineage specification of granulocyte-monocyte progenitors (GMPs) (105).

Systemic inflammation, indicated by elevated levels of TNFα, IL-1β, IL-6, and IFNγ in the serum, is clinically present in patients with periodontitis (66, 67). Plasma IFNα, a type 1 IFN, is also higher in periodontitis patients compared to healthy controls (106). However, the role of type 1 IFN-mediated trained granulopoiesis in inducing hyper-responsive neutrophils in periodontitis patients needs to be addressed experimentally. Recent evidence indicates an increased level of serum G-CSF in ligature-induced periodontitis mice (107). Fibroblasts in the periodontal tissue contribute to the release of G-CSF during periodontal inflammation, resulting in the promotion of granulopoiesis (108). Based on the cumulative evidence of the effect of cytokines and growth factors on HSPCs and the abovementioned clinical studies, it could be shown that periodontitis-associated systemic inflammation may modulate HSPCs toward trained granulopoiesis (**Table 1**). This notion was recently confirmed in a preclinical model. Specifically, it was shown that ligature-induced periodontitis (LIP)-associated systemic inflammation leads to maladaptive innate immune training in the bone marrow (i.e., generating inflammatory memory) and the generation of increased numbers of hyper-responsive neutrophils; these populate oral and non-oral tissues and promote the emergence of inflammatory comorbidities, as exemplified by the periodontitis-arthritis axis (99). This is consistent with available clinical observations as outlined below. Intriguingly, the transplantation of bone marrow from LIP-subjected mice to healthy recipient mice resulted in increased severity of arthritis in the latter, as compared with the transplantation of bone marrow from periodontally healthy mice (99). The implication of this finding (if a similar phenomenon is confirmed in humans) is that clinicians should take inflammatory memory in the bone marrow into consideration when selecting appropriate donors for hematopoietic transplantation (99).

**Table 1:** Summary of circulating molecules that are elevated in periodontitis and involved in hematopoietic tissue adaptation.

No.	Molecules	Action on hematopoietic tissue adaptation	References
1.	IL-1β	Promotes myeloid differentiation and self-renewal of HSPCs	(95, 97–99)
2.	TNF-α	Promotes survival of HSPCs and myeloid differentiation Induces myeloid progenitor apoptosis	(100)
3.	IL-6	Enhances myelopoiesis by elevating MPPs and CMPs	(101)
4.	Type 1 IFN	Mediates trained granulopoiesis with hyper-responsive neutrophils	(102)
5.	IFN-γ	Promotes HSC self-renewal and myeloid differentiation	(103)
6.	G-CSF	Drives granulopoiesis Promotes GMP expansion and granulocyte lineage specification	(104) (105)

Indeed, the notion that periodontitis might trigger an adaptation of HSPCs is supported by clinical imaging studies using ^18^F-fluorodeoxyglucose positron emission tomography/computed tomography (^18^F-FDG-PET/CT). One study revealed that periodontal inflammation was associated with hematopoietic activity in the bone marrow and arterial inflammation. Furthermore, the authors used mediation path analysis to show that the relationship between periodontal and arterial inflammation was significantly mediated by bone marrow activity (109). More recently, another study using the same cohort of 304 participants as the aforementioned study showed that periodontal inflammation (as determined by ^18^F-FDG-PET/CT) is independently correlated with not only increased arterial inflammation but also increased an risk of future cardiovascular events (64). Additionally, another study harnessing the same imaging technique also reported a trend for increased periodontal inflammation and femur bone marrow activity in patients with periodontitis (relative to controls), albeit no differences were observed in vascular inflammation between the two groups (110). The lack of clear differences in this study is likely attributed (a) to the small sample size (14 participants as opposed to >300 participants in the above-discussed studies) and (b) to the fact that participants with severe periodontal disease were under supportive periodontal therapy, which could have mitigated inflammation and associated parameters, including surrogate markers of ASCVD (discussed below) (110). Moreover, the control group also included individuals with mild periodontitis, which could also impact systemic inflammation and hematopoietic tissue activity, thus reducing potential differences compared with the experimental group.

### Alteration of Circulating Neutrophils in Periodontitis and its Putative Effect on Atherosclerosis

Patients with periodontitis exhibit elevated numbers of neutrophils and altered phenotypes presenting hyper-reactive features in cellular functions (discussed below) (21–23, 68, 69, 110–117). These include increased levels of ROS production in response to fMLP, PMA, or periodontal pathogens (21–23, 102, 111–115), elevated TNFα production following stimulation with the periodontal pathogen, *Fusobacterium nucleatum*, *in vitro* (116), and elevated neutrophil elastase levels linked to periodontal tissue destruction (117, 118). The systemic effect of this alteration has been recently confirmed in an experimental study with mice and experimental periodontitis that exhibited hyper-inflammatory neutrophil response following the exposure to secondary peritonitis compared with mice without periodontitis (119).

These altered features in neutrophils present a hallmark of trained myelopoiesis, which includes increased numbers of myeloid cells with enhanced inflammatory responsiveness (24, 95). As such, these changes may implicate the response of future inflammatory stimuli that drive certain pathological processes such as atherosclerosis.

Neutrophils that are increased in number and hyper-responsiveness due to periodontitis can contribute to any stage of this ASCVD pathology. Further research should address this hypothesis experimentally. However, it is still interesting to speculate that periodontitis-induced neutrophil alteration contributes to the association between periodontitis and ASCVD because of several clinical studies that support this notion. An elevated number of neutrophils in peripheral blood may increase the risk of ASCVD in periodontitis patients because neutrophil counts from peripheral blood are positively correlated with ASCVD risk (120, 121). Moreover, periodontitis patients consistently present with endothelial dysfunction, which is a key feature of ASCVD (2). This disturbed vascular function and elevated neutrophil ROS production triggered by periodontitis can aggravate the initiation of atherosclerosis. The latter may also potentiate the progression of atherosclerosis, particularly in oxidizing LDL. Hyper-responsiveness of neutrophils characterized by excessive production of neutrophil elastase and ROS in periodontitis patients could also contribute to the late stage of atherosclerosis as both hyper-reactive features induce endothelial apoptosis, resulting in endothelial desquamation (plaque erosion), fibrous cap thinning and plaque ruptures. Finally, whereas the production of NETs by circulating neutrophils has been shown to be comparable between patients with periodontitis and healthy controls, plasma NET degradation was lower in patients with periodontitis than in controls, suggesting an impaired NET degradation process in the plasma of periodontitis patients (122). This impairment might favor atherosclerosis complications, especially in post-ischemic stroke where NET accumulation promotes thrombus formation and expands stroke volume. In this context, a case-control study revealed that periodontitis was an independent predictor of poor outcome in post-ischemic stroke patients (123).

## Impact of Periodontitis Treatment on Neutrophils

Whereas the treatment of periodontitis improves the surrogate markers of ASCVD, its long-term cardiovascular-protective effect is uncertain. A reduction of several serum inflammatory markers that is expected to reduce the risk of ASCVD is not observed in serum IFNγ and IL-10 because both markers remain unchanged following periodontitis treatment (40). As these markers are predominantly generated and released into blood circulation by inflammatory cells, it is interesting to speculate that while successful treatment indeed achieves the resolution of periodontal inflammation, circulating inflammatory cells still retain their periodontitis-induced altered phenotypes. The latter is supported by clinical studies that investigated the functions of peripheral blood-derived neutrophils and monocytes in periodontitis patients after the treatment. The hyper-responsiveness of these myeloid cells persisted as the levels of cytokines produced by the cells in response to pathogen or LPS stimulation were comparable between neutrophils from patients before and after periodontal treatment (31, 116). Similarly, a longitudinal study on the circulating neutrophil profiles of patients with periodontitis showed that neutrophils (as a proportion of total cells isolated from peripheral blood of periodontitis patients) did not change between baseline (before treatment) and after 3-, 6-, and 12-month post-periodontitis treatment (124). The retained neutrophil phenotypes might be because of the trained myelopoiesis induced by periodontitis-triggered systemic inflammation. In murine experiments, long-lasting changes in myelopoiesis were observed following either microbial- or sterile-induced inflammation (29, 95). Similarly, a recent report shows that upon the LIP resolution, HSPCs in the bone marrow retain a myeloid differentiation bias (99).

## Targeting Neutrophils as a Novel Therapeutic Approach: Dual Benefit on Periodontitis and ASCVD

The targeted therapeutic approach for ASCVD has been established by focusing on the reduction of inflammation (**Table 2**). The first clinical trial, CANTOS, in an attempt to close the gap between pre-clinical studies and clinical practice, provided evidence that reducing inflammation could be relevant to treating atherosclerosis in humans. In this trial, the anti-IL-1β antibody, Canakinumab, was administered subcutaneously to individuals with a sustained acute myocardial infarction. The trial revealed a significant reduction in the rates of recurrent cardiovascular events, hospitalization for heart failure, and heart failure-associated death (125, 126). However, the adverse event in the group receiving Canakinumab was more significant death due to infection or sepsis compared to the placebo group (125). Moreover, oral administration of colchicine in patients with a recent myocardial infarction significantly reduces the risk of ischemic cardiovascular events (127). The benefits of colchicine were also observed in patients with chronic coronary disease (128). Unfortunately, patients in the colchicine group showed higher incidents of pneumonia and non-cardiovascular-caused death than those in the placebo group (127, 128). Other clinical studies on targeting inflammation in ASVD treatment have been reviewed elsewhere (129). These clinical trials indicate that therapies with alternative strategies are required to achieve outcomes in which the benefits outweigh the risks.

**Table 2:** Summary of clinical trials.

No.	Name of drug	Mechanism of action	Phase	Identifier (Trial registration)	Outcome
1.	Canakinumab	Binds to IL-1β resulting in blocking the interaction between IL-1β and IL-1 receptor	Phase 3	NCT01327846	Reduction in cardiovascular events, hospitalization for heart failure, and heart failure-associated death Emergence of death due to sepsis
2.	Colchicine	Prevents microtubule formation resulting in tubulin disruption	Phase 3	NCT02551094	Reduction in ischemic cardiovascular events Increase in pneumonia
3.	Metoprolol	Attenuates neutrophil migration and infiltration by impairing the neutrophil-platelet interaction that is crucial during early phases of neutrophil recruitment	Phase 4	NCT01311700	Reduction of infarct size Improvement of cardiac function
4.	AZD5069 (a CXCR2 antagonist)	Prevents neutrophil recruitment to the site of inflammation	Phase 1 Phase 2	NCT01480739 ISRCTN48328178	No adverse events and safety concerns Currently on going
5.	AMY-101 (a complement C3 inhibitor)	Inhibits downstream activation of the anaphylatoxin C3a and C5a receptors	Phase 2a	NCT03694444	Reduction in gingival inflammation Reduction of MMP-8 and MMP-9 levels in gingival crevicular fluids

Besides the necessity of different strategies to reduce the risk–benefit ratio, a refined understanding of the potential role of periodontitis-induced neutrophil alteration in the pathology of ASCVD and its retained phenotypes following successful periodontitis treatment highlights the importance of selectively targeting neutrophils during the treatment of periodontitis. This therapeutic approach is warranted to mitigate the potential impact of periodontitis on ASCVD. The main endpoint of this intervention is to either manipulate the number of neutrophils and/or their functional activities. We only discuss the approaches to block neutrophil recruitment and prevent NET-driven inflammation, while other neutrophil-targeted therapeutics have been extensively reviewed elsewhere (130).

While the results of neutrophil recruitment blockage are promising in preclinical studies, clinical trials exhibit unsuccessful outcomes due to the redundancy of signals during neutrophil recruitment and off-target effects caused by receptor cross-linking. Recent studies have provided strategies for overcoming such difficulties. The combined inhibition of several endothelial cell molecules interrupts redundant signals that recruit neutrophils (131). Intravenous injection of nanoparticles carrying small interfering RNAs (siRNAs) that target endothelial adhesion molecules including ICAM-1, E-, P-selectin, and vascular cellular adhesion molecule-1 (VCAM-1) decreased leukocyte recruitment to ischemic myocardium in a mouse model of post-myocardial infarction (131). Moreover, specific blockage of neutrophils that traffic to certain vascular tissue requires a refined understanding of recruitment patterns at particular sites (16). A neutrophil granule protein, cathepsin G promotes myeloid cell adhesion to only arterial but not microvascular tissue (73). Indeed, antibody-assisted cathepsin G neutralization in an atherosclerotic mouse model specifically alleviated neutrophil recruitment to the carotid artery, resulting in reduced atherosclerotic plaque size. However, a similar treatment did not affect neutrophil adhesion in lung microcirculation following an LPS-induced lung inflammation model in mice (73). Furthermore, the elucidation of heteromeric interactions between neutrophil-derived human neutrophil peptide-1 (HNP1) and platelet-borne CCL5 that must stimulate monocyte adhesion *via* CCR5 ligation allows the design of a peptide that can disturb these neutrophil–platelet interactions. This specifically designed short peptide alleviated inflammation in a mouse model of myocardial infarction (20). Finally, inducing endogenous inhibitors in leukocytes can overcome integrin activation by chemokine, thereby suppressing myeloid cell adhesion. For example, growth differentiation factor-15 (GDF-15) and annexin A1 inhibit chemokine-induced β2 integrin activation and subsequently reduce neutrophil recruitment in a mouse model of chronic inflammation (72, 132). Moreover, recombinant developmental endothelial locus-1 (DEL-1), the first identified endogenous inhibitor of the leukocyte adhesion cascade (133), inhibited neutrophil recruitment in mouse and non-human primate models (17, 134).

Important roles of NETs in both atherosclerosis progression (atherogenesis, plaque destabilization and erosion) and complication (atherothrombosis) stand out as a potential therapeutic target to manipulate cardiovascular inflammation. Peptidyl arginine deiminase 4 (PAD4) citrullinates histone to disrupt electrostatic bonds in nucleosomes, decondensing chromatin that leads to NET release (135). Cl-amidine, a PAD inhibitor administered intravenously in an atherosclerotic mouse model, prevented NET formation, leading to reduced atherosclerotic lesion area and thrombosis (136). However, the mechanism of NETosis in mice is different from that in humans as *ex vivo* experiments of human neutrophils showed that PMA-induced NETosis of the cells was not affected following the PAD4 inhibitor, Cl-amidine (137). Meanwhile, DNase 1 treatment might be considered to mitigate ASCVD complications like thrombosis due to NET deposition in the vascular lumen. This notion is supported by a study where the treatment protected mice from deep vein thrombosis following an inferior vena cava stenosis model (138). A reduction in lesion size was also observed in an atherosclerosis mouse model after DNase injections (84). In humans, DNAse 1 treatment to eliminate NET deposition might need a combination with other substances, as DNAse 1 alone was not adequate to degrade NETs *in vitro* (139). Lastly, NET chromatin can stimulate macrophages by activating AIM2 inflammation, causing the release of IL-8 and IL-1β in atherosclerotic lesions. The use of an AIM2 inhibitor could also attenuate ASCVD complications because reduced plaque vulnerability through the thickening of the fibrous cap was observed in an atherosclerotic mouse model after the treatment of an AIM2 inhibitor (140). ApoE-deficient mice on a 6-week high fat diet and injected with anti-chromatin antibodies also showed a reduced plaque area per lumen, suggesting the potential of chromatin blockage to hinder atherosclerosis (141). The use of substances to block NETs in humans should be implemented with caution because studies about the pro-inflammatory features of NETs in humans are still conflicting, as *in vitro* NET clearance by human macrophages did not induce pro-inflammatory cytokines (139), but NET transfection to mouse macrophages did (142).

Targeting neutrophils as an approach to mitigate the potential impact of periodontitis on ASCVD is appealing considering the numerous efforts outlined previously. This targeted strategy can not only have direct effects on ASCVD but also reduce periodontitis, which triggers inflammation that primes neutrophils for ASCVD pathology. However, safety and specificity issues are challenges that need to be solved as neutrophils interact with other myeloid lineages (130). The solution to the former concern is that the intervention should consider the therapeutic window, where the attenuation of the inflammatory process mediated by neutrophils does not interfere with neutrophil capacity during host defense (130). Recent studies revealed that the use of antibodies to inhibit NET-derived histones reduced the amplification of NET-induced inflammation rather than completely blocking NETosis or inflammation (141). Few studies have presented strategies to obtain the specificity to therapeutically target neutrophils. For example, the conjugation of siRNA targeting Bruton’s tyrosine kinase (BTK) to the F(ab’)2 fragment of an anti-neutrophil monoclonal antibody specifically targeted alveolar neutrophils in an acute lung injury mouse model. This treatment was administered locally using a technique called intranasal instillation (143). Meanwhile, others harness nanoparticle technology to enhance specificity. One of them reported that neutrophils adhered to activated endothelium-engulfed albumin nanoparticles carrying piceatannol, leading to the inactivation of these adherent neutrophils and consequently preventing vascular inflammation (144). Additionally, another report exhibited lipid-based nanoparticles that were successfully incorporated with identified peptides that interact with the neutrophil-specific surface marker, CD177 (145).

A clinical trial targeting neutrophils to lower ASCVD risk is still lacking. However, two randomized clinical trials reported that metoprolol provides a cardio-protective effect following acute myocardial infarction, which is one of the atherosclerosis complications (**Table 2**). Specifically, intravenous administration of metoprolol reduced infarct size and improved cardiac function in patients with acute myocardial infarction (146, 147). Studies using a myocardial infarct mouse model revealed the mechanism of this protection in which metoprolol attenuates neutrophil migration and infiltration by impairing the neutrophil–platelet interaction that is crucial during early phases of neutrophil recruitment (148, 149). Another drug candidate, AZD5069, a CXCR2 antagonist, could be a potent drug candidate in treating patients with advanced atherosclerotic lesions. CXCR2 is a chemokine receptor 2 in neutrophils that regulates neutrophil migration, and the inhibition of this receptor successfully prevents neutrophil recruitment to the site of inflammation (150). No adverse effects on neutrophil function were observed, and no safety concerns were raised in participants receiving oral administration of AZD5069 (151). The CICADA trial was proposed to investigate the effect of a CXCR2 antagonist (administered orally) on coronary flow, structure, and function in patients with coronary heart disease (152).

Mechanical intervention in periodontitis management with adjunctive neutrophil-targeted therapeutic approach could also provide benefit in reducing periodontal-induced systemic inflammation that can implicate all stages of atherosclerosis. A phase IIa clinical trial of the complement C3 inhibitor, AMY-101, summarized in **Table 2**, shows promising results in reducing local inflammation in periodontal tissue (153). The pharmacological blockade of the central complement component, C3, inhibits downstream activation of the anaphylatoxin C3a and C5a receptors (C3aR and C5aR, respectively), which induce inflammatory bone loss in a preclinical model (154). C5aR and TLR2 coactivation in neutrophils contribute to oral microbiota dysbiosis leading to overt periodontal inflammation and subsequent periodontal tissue destruction (155). Local injection of AMY-101 to the gingiva reduced gingival inflammation without adverse events, warranting phase III clinical trials for further investigation (153, 156). Importantly, AMY-101 significantly reduced the gingival crevicular levels of MMP-8 and MMP-9 (152), which are the major neutrophil-derived proteases and are considered biomarkers of periodontal tissue destruction (157). Meanwhile, other local neutrophil-targeted treatments, including resolvin E1, developmental endothelial locus-1, and milk fat globule epidermal growth factor 8, are still in pre-clinical studies. These proteins reduce neutrophil recruitment to the site of inflammation and prevent animal models of ligature-induced periodontitis (134, 158, 159). Additionally, resolving E1 also promotes neutrophil apoptosis and its clearance (efferocytosis), resulting in the resolution of periodontal inflammation (157). Focusing on the termination of periodontitis may prevent its systemic impact, which is heightened systemic inflammation. These strategies of local intervention potentially further improve the current periodontal treatment in providing a sustained protection to cardiovascular health.

## Conclusion

**Figure 3:**
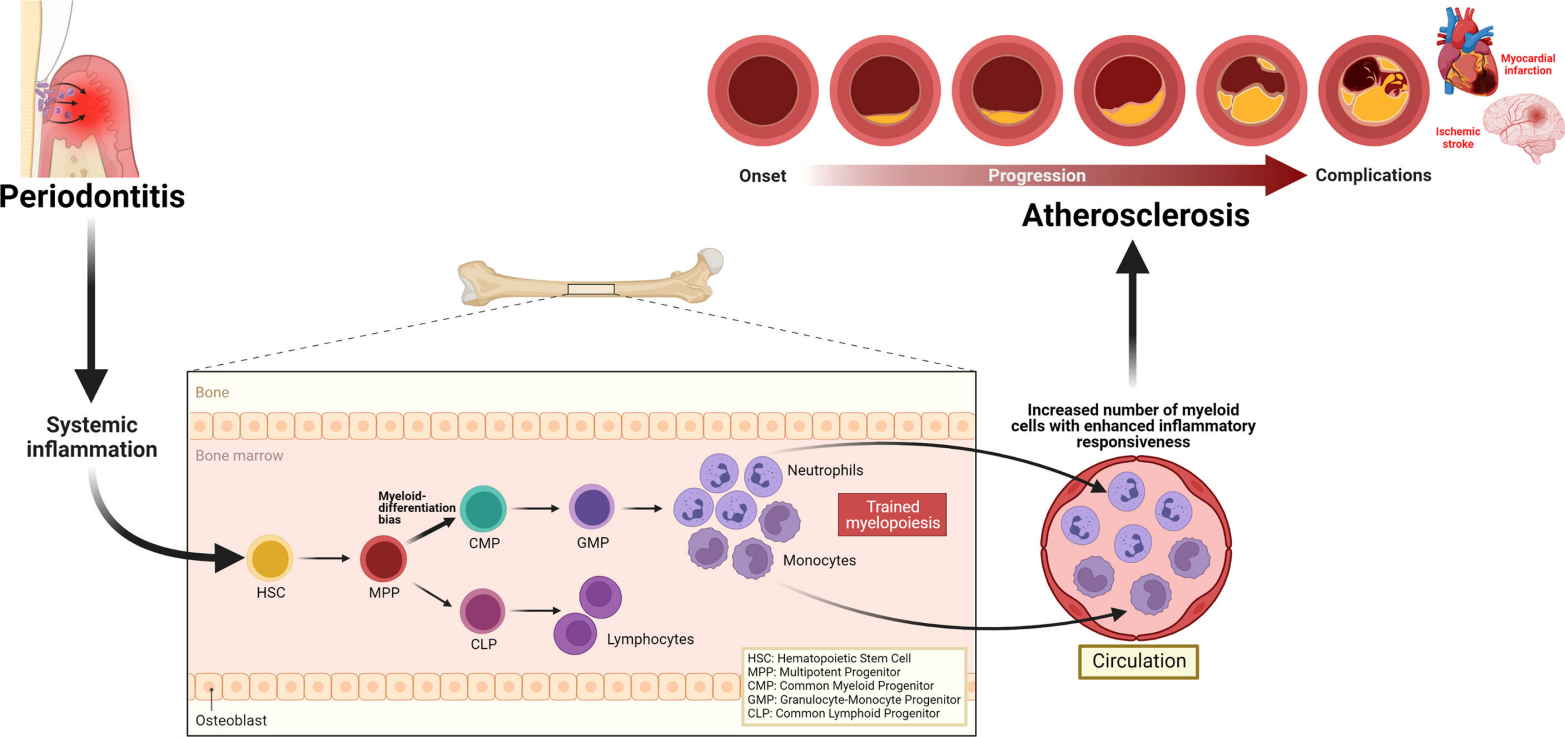
Periodontitis-induced systemic inflammation, inflammatory modulation of bone marrow progenitor cells, and implications for atherosclerosis. Periodontitis triggers systemic inflammation, which causes inflammatory modulation of hematopoietic stem and progenitor cells, resulting in a trained myelopoiesis. In turn, as a hallmark of trained myelopoiesis, an increased number of myeloid cells, including neutrophils with enhanced inflammatory responsiveness, may contribute to all stages of the ASCVD pathology, atherosclerosis.

Periodontitis triggers systemic inflammation which could modulate hematopoietic tissue activity in the bone marrow, resulting in trained myelopoiesis. Clinical studies demonstrating increased numbers as well as enhanced inflammatory responsiveness in neutrophils back this notion. The evidence of persistent elevated neutrophil numbers and their altered phenotypes, despite the resolution of periodontitis following local treatment, also supports the speculation that periodontitis-induced systemic inflammation can induce long-term myelopoiesis bias, a hallmark of innate immune training in the bone marrow. The quantitative and qualitative alterations in neutrophils may contribute to all stages of the ASCVD pathology, atherosclerosis (**Figure 3**). Although clinical intervention studies suggest that periodontal therapy improves surrogate markers of ASCVD, the long-term effects of this oral treatment to maintain such improvement and convincing evidence that successful periodontitis treatment can reduce the risk or incidence of ASCVD are yet to be investigated. Meanwhile, targeting neutrophils is warranted to improve local periodontal therapy, eliminate periodontitis effectively, that can reduce periodontitis-triggered systemic inflammation and reverse the periodontitis-induced neutrophil changes. Such targeted approaches can be harnessed as a direct treatment for ASCVD and indirect intervention of the disease through the reduction of heightened systemic inflammation triggered by periodontitis.

## Author Contributions

RI wrote the original draft. RI, SC, GH, VP, JD, and FD provided critical revisions to the article. All authors listed have made a substantial, direct, and intellectual contribution to the work and approved it for publication.

## Funding

This work was undertaken at the UCL Biomedical Research Centre which receives funding from the UK National Institute for Health and Care Research. GH receives a grant from the US National Institutes of Health (DE031206). RI receives funding from the Indonesia Endowment Fund for Education (S-1692/LPDP.4/2019 —LPDP—Indonesia Scholarship). The figures were created using Biorender.com

## Conflict of Interest

The authors declare that the research was conducted in the absence of any commercial or financial relationships that could be construed as a potential conflict of interest.

## Publisher’s Note

All claims expressed in this article are solely those of the authors and do not necessarily represent those of their affiliated organizations, or those of the publisher, the editors and the reviewers. Any product that may be evaluated in this article, or claim that may be made by its manufacturer, is not guaranteed or endorsed by the publisher.
